# α5β1 integrin induces the expression of noncartilaginous procollagen gene expression in articular chondrocytes cultured in monolayers

**DOI:** 10.1186/ar4307

**Published:** 2013-09-19

**Authors:** Nobuho Tanaka, Yasuko Ikeda, Tetsuo Yamaguchi, Hiroshi Furukawa, Hiroyuki Mitomi, Takumi Nakagawa, Shigeto Tohma, Naoshi Fukui

**Affiliations:** 1 Clinical Research Center, National Hospital Organization Sagamihara Hospital, Sakuradai 18-1, Sagamihara, Kanagawa 252-0315, Japan; 2 Department of Human Pathology, Juntendo University, Hongo 2-1-1, Bunkyo-ku, Tokyo 113-8421, Japan; 3 Department of Orthopaedic Surgery, School of Medicine, Teikyo University, Kaga 2-11-1, Itabashi-ku, Tokyo 173-8605, Japan; 4 Graduate School of Arts and Sciences, The University of Tokyo, Komaba 3-8-1, Meguro-ku, Tokyo 153-8902, Japan

## Abstract

**Introduction:**

Articular chondrocytes undergo an obvious phenotypic change when cultured in monolayers. During this change, or dedifferentiation, the expression of type I and type III procollagen is induced where normal chondrocytes express little type I and type III procollagen. In this study, we attempted to determine the mechanism(s) for the induction of such procollagen expression in dedifferentiating chondrocytes.

**Methods:**

All experiments were performed using primary-cultured human articular chondrocytes under approval of institutional review boards. Integrin(s) responsible for the induction of type I and type III procollagen expression were specified by RNAi experiments. The signal pathway(s) involved in the induction were determined by specific inhibitors and RNAi experiments. Adenovirus-mediated experiments were performed to identify a small GTPase regulating the activity of integrins in dedifferentiating chondrocytes. The effect of inhibition of integrins on dedifferentiation was investigated by experiments using echistatin, a potent disintegrin. The effect of echistatin was investigated first with monolayer-cultured chondrocytes, and then with pellet-cultured chondrocytes.

**Results:**

In dedifferentiating chondrocytes, α5β1 integrin was found to be involved in the induction of type I and type III procollagen expression. The induction was known to be mediated by v-akt murine thymoma viral oncogene homolog (AKT) signaling. Among the three AKT isoforms, AKT1 seemed to be most involved in the signaling. Elated RAS viral (r-ras) oncogene homolog (RRAS) was considered to regulate the progression of dedifferentiation by modulating the affinity and avidity of α5β1 integrin to ligands. Echistatin inhibited dedifferentiation of monolayer-cultured chondrocytes. Furthermore, the matrix formed by pellet-cultured chondrocytes more closely resembled that of normal cartilage compared with the controls.

**Conclusions:**

The result of this study has shown, for the first time, that α5β1 integrin may be responsible for the induction of non-cartilaginous collagen expression in chondrocytes undergoing dedifferentiation. Again, this study has shown that the inhibition of ligand ligation to integrins may be an effective strategy to inhibit phenotypic change of cultured chondrocytes, and to improve the quality of matrix synthesized by primary cultured chondrocytes.

## Introduction

Articular chondrocytes undergo an obvious phenotypic change when isolated from cartilage matrix and cultured in a monolayer. During this change, or dedifferentiation, the cell metabolism obviously changes, and the matrix synthesized by the cells changes from one unique cartilage to another similar to that generated by fibroblasts [1,2]. Residing within cartilage matrix, chondrocytes express cartilage matrix components such as type II collagen and aggrecan, but synthesize little type I or type III procollagen, which are trace components of normal articular cartilage. With the initiation of dedifferentiation, the expression of type II collagen and aggrecan declines gradually, and the expression of type I and type III procollagens is induced instead. In parallel with this metabolic change, the cell shape changes dramatically from the original spherical form to flattened elongated forms resembling those of fibroblasts [1].

Although dedifferentiation is a critical problem in tissue engineering [3-5], the exact mechanism(s) for dedifferentiation has not been known for decades. In a recent study, we reported that αvβ5 integrin may play a critical role in dedifferentiation [6]. In monolayer-cultured chondrocytes, αvβ5 integrin suppresses the expression of cartilage matrix components through the activation of Elk-related tyrosine kinase (ERK) signaling, and promotes morphological change of the cells. However, in that study αvβ5 integrin was found not to be involved in the induction of type I or type III procollagen expression. The mechanism for the appearance of these noncartilaginous procollagens thus remains unknown.

In the present study, we attempt to elucidate this mechanism for the induction of type I and type III procollagen expression in monolayer-cultured chondrocytes. Through a series of experiments, we obtained results indicating that α5β1 integrin may be a key molecule for the induction. We also found that the inhibition of ligand ligation to integrins indeed prevented dedifferentiation of chondrocytes cultured in a monolayer, and improved the quality of matrix generated by pellet-cultured chondrocytes.

## Methods

### Antibodies and reagents

A function blocking anti-α5β1 integrin mouse monoclonal antibody (JBS5) was purchased from Merck Millipore (Billerica, MA, USA). Rabbit polyclonal anti- related RAS viral (r-ras) oncogene homolog (anti-RRAS) antibody and mouse control IgG were obtained from Santa Cruz Biotechnology (Santa Cruz, CA, USA), and phosphospecific and nonspecific antibodies for v-akt murine thymoma viral oncogene homolog (AKT; Thr308 and Ser473) and ERK were obtained from Cell Signaling Technology (Danvers, MA, USA). Anti-type I collagen rabbit polyclonal antibody was purchased from ThermoFisher Scientific (Waltham, MA, USA). SB202190, SB203580, PD98059, U0126, Wortmannin, LY294002, Akt Inhibitor IV and Akt Inhibitor VIII were from Merck Millipore. SP600125, GF1009203X and echistatin were obtained from Sigma (St Louis, MO, USA). Bovine fibronectin and bovine serum albumin (fraction V; BSA) were also obtained from Sigma. CP4715 was a kind gift from Meiji Seika Pharma (Tokyo, Japan).

### Cartilage and chondrocyte culture

The study was performed under the approval of the institutional review boards of National Hospital Organization Sagamihara Hospital, JR Tokyo General Hospital, and International Medical Center of Japan. Informed consent was obtained in writing from all patients who offered cartilage.

Human articular cartilage was obtained from the macroscopically preserved areas within osteoarthritic knee joints during prosthetic surgery. Primary cultured human articular chondrocytes were prepared from those cartilages by serial enzymic digestion using Pronase (Merck Millipore, Darmstadt, Germany) and Collagenase P (Roche Diagnostics, Rotkreuz, Switzerland) [7]. Following digestion, chondrocytes were plated onto polystyrene culture dishes (Iwaki, Chiba, Japan) at a density of 2 × 10^5^/cm^2^, and maintained in Dulbecco’s modified Eagle’s medium/F-12 containing 10% fetal bovine serum and 25 μg/ml ascorbic acid. For pellet culture, 1 × 10^6^ chondrocytes were placed in a 1.5 ml polyethylene centrifuge tube, which was centrifuged at 200 × *g* for 5 minutes to form a pellet at the bottom. The pellets were maintained in the media used for the monolayer culture.

### RNA interference

All siRNAs were obtained from Qiagen (Hilden, Germany). Sequences for these siRNAs are provided in Additional file 1. siRNAs were introduced into primary cultured chondrocytes by electroporation using a Nucleofector (Lonza, Basel, Switzerland), following the manufacturer’s protocol with some modifications [7]. For each gene, two or three siRNAs were used to suppress the expression, which was confirmed by quantitative PCR. The suppression of RRAS expression was also confirmed at the protein level by western blotting, while the suppression of expression of α5, α10, α11, αv, β1, β5 and β8 integrins was validated by flow cytometry.

### Generation of recombinant adenoviruses

Recombinant adenoviruses carrying constitutively active (CA) or dominantly negative (DN) mutants of *HRAS*, *RRAS*, *RAP1A*, *RAP1B*, and *CDC42* were generated using a ViraPower Adenovisal Expression System (Life Technology, Grand Island, NY, USA) as described before [7]. In brief, human *HRAS*, *RRAS*, *RAP1A*, *RAP1B*, and *CDC42* complementary DNA were cloned into the adenoviral-generating constructs after the introduction of CA or DN mutations. These constructs were then transfected into 293A cells (Life Technology) using FuGENE 6 (Roche Diagnostics), and the cells were subcultured to generate recombinant adenoviruses carrying these genes under the control of the human cytomegalovirus immediate-early enhancer/promoter. The viruses were titrated by limiting dilution plaque titration on 293A cells, and used at 50 to 100 plaque-forming units/cell. In preliminary experiments, the efficiency of transduction by this method was confirmed to be almost 100%.

### Cell attachment assay

A cell attachment assay was performed based on a previously described method [6,8]. In brief, primary cultured human chondrocytes were prepared and maintained in a monolayer as described earlier. For assay, the cells were harvested and suspended in serum-free media at a density of 1 × 10^6^ cells/ml. After a 90 minute recovery time, 100 μl cell suspension was placed in each well of a 96-well microtiter plate (Iwaki), some wells of which were precoated with fibronectin or BSA. Cells were allowed to attach to the plates for 60 minutes at 37°C. The unattached cells were then removed by gentle washing, and the numbers of cells bound to the plates were estimated by the amounts of DNA in respective wells, which were determined by the Quant-iT dsDNA Assay Kit (Life Technology).

### Western blot analysis

For Western blot analysis, cell lysate was obtained from cultured chondrocytes and clarified by centrifugation. Protein concentration was determined by the Pierce BCA Protein Assay kit (ThermoFisher Scientific), and 20 μg protein was subjected to SDS-PAGE and transferred onto a nitrocellulose membrane. After blocking, the membrane was incubated with a primary antibody and then with an appropriate secondary antibody conjugated with peroxidase (Santa Cruz Biotechnology). In this study, all primary antibodies were used at the concentration of 1 μg/ml. Immunoreactive protein was finally visualized using a SuperSignal West Pico chemiluminescent substrate (ThermoFisher Scientific). For some samples, band densities were quantified by ImageJ image analysis software (version 1.46; NIH, Bethesda, MD, USA).

### Pull-down assay

The amount of active RRAS protein was determined by a pull-down assay using a GST fusion protein of the RAS-binding domain of RAF1 (GST-Raf1-RBD; ThermoFisher Scientific) and subsequent western blot analysis [6]. The amount of total RRAS in the same lysate was determined by western blot analysis.

### Immunofluorescence staining

Formation of focal adhesion and filamentous actin assembly was evaluated by fluorescence microscopy. In this experiment, the cells were fixed with 4% paraformaldehyde in PBS and permealized with 0.1% Trixon X-100. After blocking with 1% BSA in PBS, the cells were first incubated with an anti-vinculin mAb (10 μg/ml, clone 7 F9; Millipore) and then a tetramethylrhodamine isothiocyanate-conjugated anti-phalloidin rabbit polyclonal antibody (8 μg/ml; Millipore). The former antibody was visualized using a fluorescein isothiocyanate-conjugated secondary antibody (10 μg/ml, AP124F; Millipore). After staining, cells were observed under a fluorescence microscope (Olympus BX51; Olympus, Tokyo, Japan).

### Evaluation of sulfated proteoglycan synthesis

Quantitative assessment of proteoglycan synthesis in pellet-cultured chondrocytes was performed by a previously described method [7]. In brief, the culture medium was replaced with a fresh one containing 0.1% fetal bovine serum and 10 μCi/ml [^35^S]sulfate. After 4 hours of labeling, a pellet was recovered, rinsed extensively with ice-cold PBS, and subjected to papain digestion at 55°C for 16 to 24 hours with gentle agitation. The digest was centrifuged and the radioactivity of the supernatant was measured. The radioactivity was normalized by the DNA content of the supernatant, which was determined by the Quant-iT dsDNA Assay Kit (Life Technology).

### Histological evaluations

For histological evaluations, chondrocyte pellets were fixed in paraformaldehyde, embedded in paraffin, and sections 6 μm thick were prepared. The sections were stained with hematoxylin and eosin, or Safranin O and fast green, and were observed under a light microscope. For immunohistochemistry, the sections were digested with 1.0% hyaluronidase (Sigma) for antigen retrieval, and then incubated overnight with an anti-type I collagen polyclonal antibody prepared at the concentration of 2 μg/ml in PBS. The antibody was finally visualized with the avidine-linked peroxidase system (Santa Cruz Biotechnology) coupled with 3-amino-9-ethylcarbazole substrate (Dako, Glostrup, Denmark).

### Statistics

Data were analyzed by paired *t* test or repeated-measures one-way factorial analysis of variance (repeated-measures analysis of variance). If the analysis of variance showed significance, data were further analyzed by Fisher’s protected least-significant difference test as a *post hoc* test. The level of significance was set at *P* <0.05.

## Results

### α5β1 integrin may mediate induction of noncartilaginous procollagen gene expression in monolayer-cultured chondrocytes

**Figure 1:**
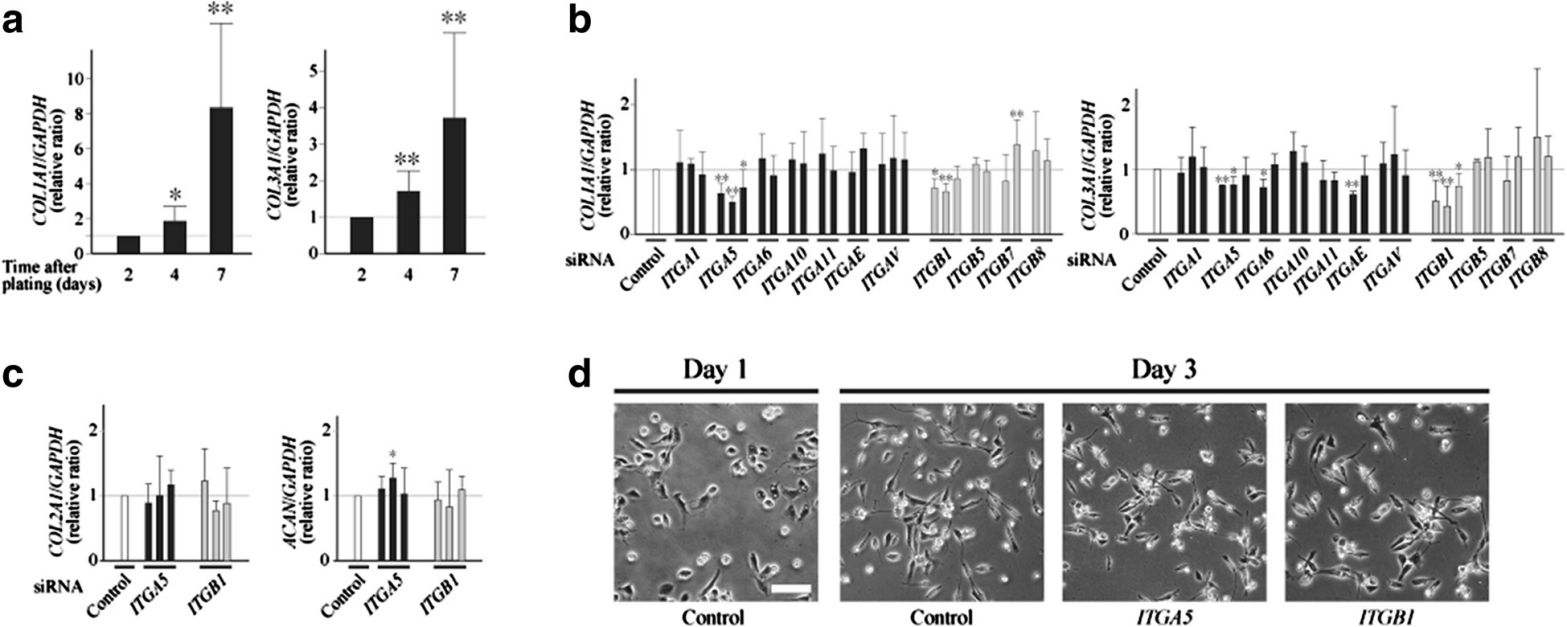
**α5β1 integrin may be involved in the induction of noncartilaginous collagen expression in dedifferentiating chondrocytes. (a)** Chondrocytes obtained from human articular cartilage were cultured in monolayers, and expression of type I procollagen (*COL1A1*) and type III procollagen (*COL3A1*) was determined by quantitative PCR at the indicated time points. Change in expression level during culture is shown by relative ratios against that at day 2. Results are mean ± standard error of the mean (SEM) of four independent experiments, each in triplicate. **(b)**, **(c)** siRNAs for respective integrins were introduced into primary cultured chondrocytes, and cells were cultured in monolayers. Three days later, expression of *COL1A1* and *COL3A1* was determined by quantitative PCR **(b)**. For some siRNAs, expression of type II procollagen (*COL2A1*) and aggrecan (*ACAN*) was also determined **(c)**. Results shown by relative ratios against the cells given control siRNA (Control; open bars). Two or three bars for respective integrin genes indicate the results of respective siRNAs used for the genes. Results are mean ± SEM of four to six independent experiments, each in triplicate. *P <0.05 and **P <0.01 against day 2 or control. **(d)** Control siRNA or siRNA for α5 or β1 integrin was introduced into chondrocytes, and the cells were cultured in monolayers. Cell morphology was observed 1 day (day 1) or 3 days (day 3) after introduction. Representative images of six independent experiments are shown. Scale bar = 100 μm. GADPH, glyceraldehyde 3-phosphate dehydrogenase.

First, the expression of type I and type III procollagen was evaluated sequentially for 1 week in primary cultured human articular chondrocytes maintained in monolayers. In those cells, the expression of both procollagen genes increased dramatically after plating, confirming the results of previous studies [9-11] (Figure 1a). Of these two genes, the increase was more obvious with type I procollagen (*COL1A1*), which showed a nearly eightfold increase in the first 7 days after plating. In the following part of this study, we attempted to clarify the mechanism(s) for this induction of noncartilaginous procollagen gene expression.

Previously, we determined 11 dominant integrins in human articular chondrocytes [6]. To examine the involvement of respective integrins in the induction of type I or type III procollagen expression, we suppressed the expression of those 11 dominant integrins one by one by RNAi, and observed whether any change occurred in the expression levels of the procollagen expression. In this experiment, the suppression of α5 or β1 integrin expression resulted in significant reduction of type I and type III procollagen expression (Figure 1b), while their suppression did not alter the expression of type II procollagen or aggrecan (Figure 1c). An MTT assay confirmed that cell viability was little affected by the introduction of siRNAs for either integrin gene (Additional file 2). We then examined whether the change of cell shape after plating was affected by RNAi for α5 or β1 integrin, and confirmed our previous observation that these integrins were unlikely to be involved in the change of cell morphology [6] (Figure 1d). α5 and β1 integrins form a functional heterodimer on a cell [12]. These results thus suggest a possibility that α5β1 integrin may promote the induction of type I and type III procollagen expression in dedifferentiating chondrocytes.

### α5β1 integrin induces noncartilaginous procollagen gene expression through the activation of PI3K/AKT signaling in dedifferentiating chondrocytes

**Figure 2:**
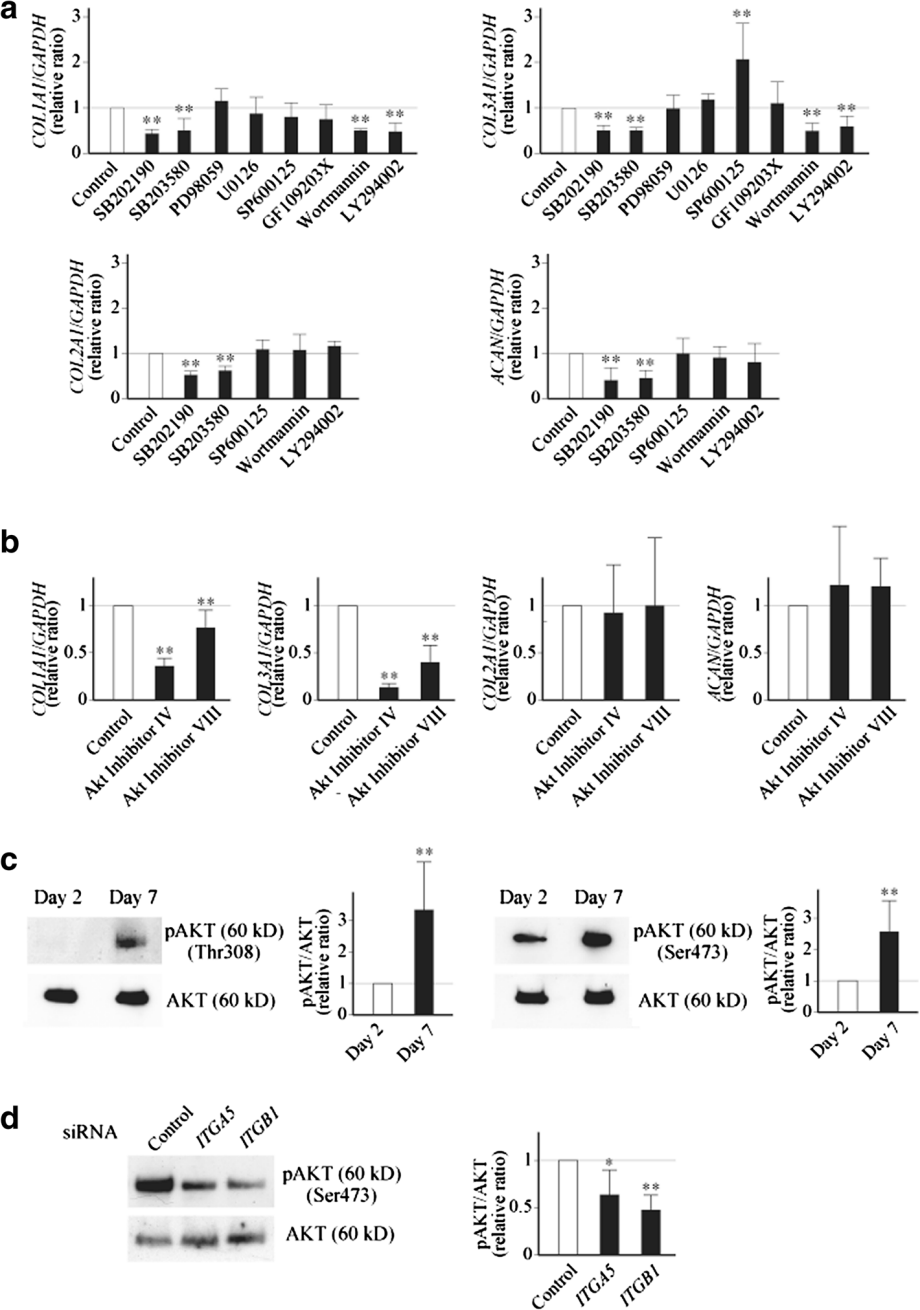
**Phosphoinositide 3-kinase/AKT signaling may be involved in induction of noncartilaginous collagen expression in dedifferentiating chondrocytes. (a)** Primary cultured chondrocytes maintained in monolayers for 5 days were treated with specific signal inhibitors for 24 hours, and expression of type I procollagen (*COL1A1*) and type III procollagen (*COL3A1*) was evaluated by quantitative PCR. For some cells, expression of type II procollagen (*COL2A1*) and aggrecan (*ACAN*) was also evaluated. **(b)** Experiment was repeated using two specific inhibitors for AKT phosphorylation, and expression of the above four genes was evaluated by quantitative PCR. **(a)**, **(b)** Inhibitors were used at following concentrations: SB202190, 20 μM; SB203580, 20 μM; PD98059, 20 μM; U0126, 10 μM; SP600125, 10 μM; GF109203X, 5 μM; Wortmannin, 0.5 μM; LY294002, 20 μM; Akt inhibitor IV, 5 μM; Akt inhibitor VIII, 5 μM. Results are shown by relative ratios against control cells treated with dimethylsulfoxide (Control; open bars). Bars represent mean ± standard error of the mean (SEM) of three **(b)**, five (SB202190, SB203580, U1026 in **(a)**) or six (the other inhibitors in **(a)**) independent experiments. **P <0.01 against control cells. **(c)** In monolayer-cultured chondrocytes, phosphorylation of AKT at Thr308 and Ser473 was evaluated 2 and 7 days after plating by western blotting, using phosphospecific (pAKT) and then nonphosphospecific anti-AKT antibodies (AKT). **(d)** Control siRNA, or siRNA for α5 or β1 integrin was introduced into chondrocytes, and the cells were cultured in monolayers. Phosphorylation of AKT at Ser473 was evaluated 3 days after plating. **(c)**, **(d)** Experiments were repeated five times, and representative results are shown together with results of densitometric measurement. For the latter, results are mean ± SEM. GADPH, glyceraldehyde 3-phosphate dehydrogenase.

When bound to ligands, an integrin heterodimer activates intracellular signaling to induce a cellular response [13,14]. We thus next attempted to determine the signal pathway(s) activated by α5β1 integrin and induces the expression of the noncartilaginous procollagens. For this, monolayer-cultured chondrocytes were treated with a panel of specific signal inhibitors, and the change in gene expression was evaluated. In this experiment, Wortmannin and LY294002, inhibitors for phosphatidylinositol 3-kinase, were found to reduce the expression of type I and type III procollagen in dedifferentiating chondrocytes, without changing the expression of type II procollagen or aggrecan (Figure 2a). The expression of type I and type III procollagen was also suppressed by SB202190 and SB203580 that inhibit p38 signaling, but these inhibitors suppressed the expression of type II collagen and aggrecan as well, indicating that p38 signaling may not be responsible for the induction of type I and type III procollagen expression during dedifferentiation. Inhibition of c-Jun N-terminal kinase by SP600125 obviously enhanced type III procollagen expression without affecting type I procollagen expression. Meanwhile, inhibition of protein kinase-C by GF109203X did not cause any significant change in the expression of either gene investigated here. From this result, phosphoinositide 3-kinase/AKT signaling was considered to be involved in the induction of the noncartilaginous procollagen expression. To examine this possibility, the experiment was repeated using two specific inhibitors for AKT phosphorylation, and consistent results were obtained (Figure 2b).

Based upon these results, we evaluated levels of AKT phosphorylation in monolayer-cultured chondrocytes at 2 and 7 days after plating, and confirmed that the phosphorylation was in fact promoted during that period (Figure 2c). Next, to demonstrate the involvement of α5β1 integrin in the elevation of AKT phosphorylation, the expression of α5 or β1 integrin was suppressed by RNAi, and the phosphorylation of AKT was evaluated. In this experiment, the phosphorylation of AKT was in fact reduced by the suppression of α5 or β1 integrin expression (Figure 2d). These results consistently support our proposed hypothesis that phosphoinositide 3-kinase/AKT signaling is promoted in dedifferentiating chondrocytes via α5β1 integrin, which induces the expression of noncartilaginous procollagens.

AKT has three isoforms in human. Thus, we finally attempted to clarify which isoform is most involved in the induction of noncartilaginous procollagen gene expression during dedifferentiation. From the results of the RNAi experiment, AKT1 was considered to play the most critical role in the induction among the three isoforms (Figure S2a,b in Additional file 3), where AKT2 might be the most abundant isoform in human articular chondrocytes (Figure S2c in Additional file 3).

### Small GTPase RRAS regulates α5β1 integrin activity and promotes noncartilaginous procollagen gene expression in dedifferentiating chondrocytes

In the previous study we have shown that in dedifferentiating chondrocytes the activity of αvβ5 integrin, or the avidity and affinity of the integrin to ligands, is regulated by a small GTPase RRAS [6]. During the course of dedifferentiation, RRAS is gradually activated, which promotes dedifferentiation process by activating αvβ5 integrin. In light of this finding, we investigated whether the activity of α5β1 integrin is also regulated by RRAS in monolayer-cultured chondrocytes.

**Figure 3:**
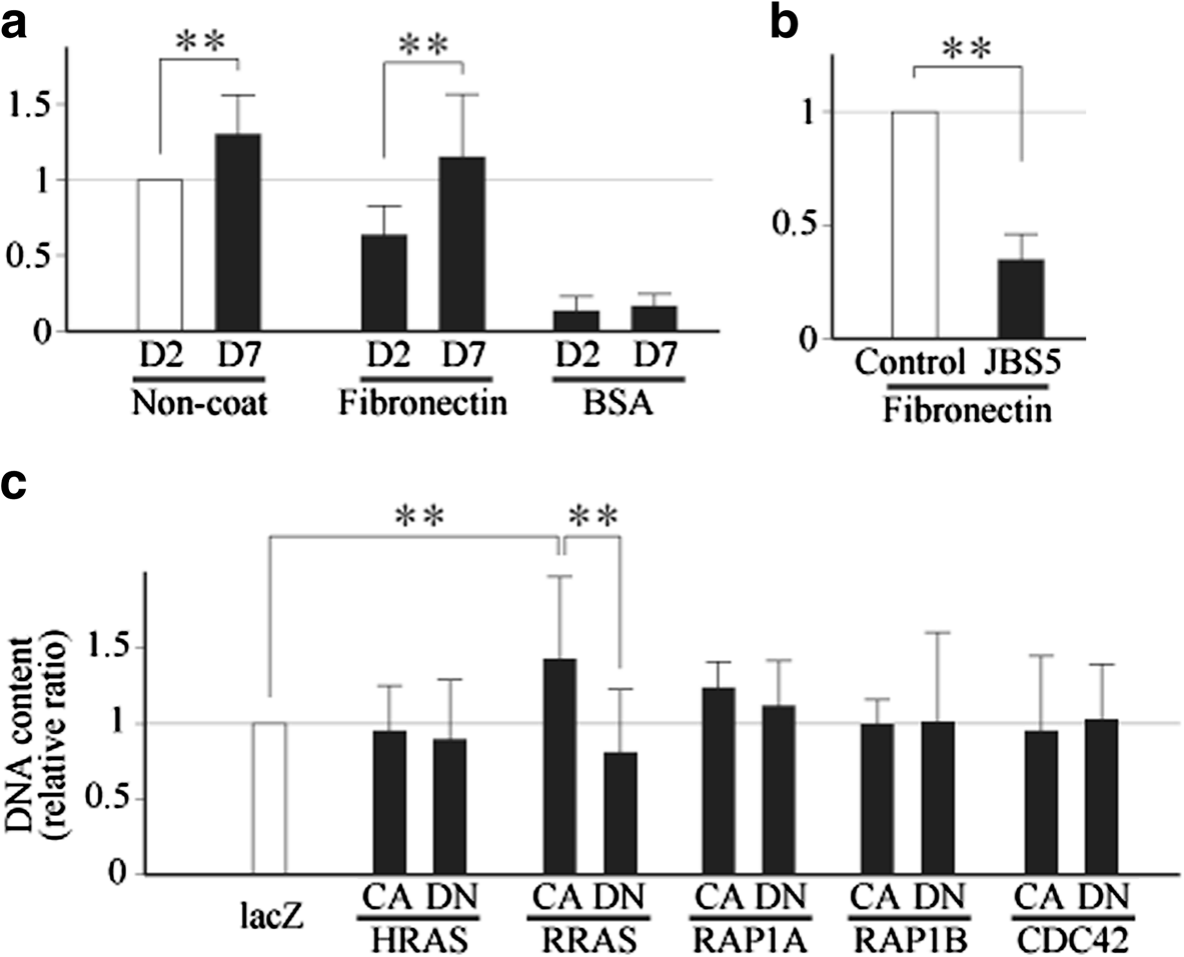
**α5β1 integrin is gradually activated in dedifferentiating chondrocytes, possibly by RRAS. (a)** Chondrocytes were cultured in monolayers for 2 days (D2) or 7 days (D7), and cell attachment was evaluated at respective time points on noncoated plates (Non-coat) or plates coated with fibronectin or BSA. **(b)** Attachment to fibronectin-coated plates was evaluated using 7-day cultured chondrocytes after incubation with a function blocking anti-α5β1 integrin antibody (JBS5; 1 μg/ml) or control IgG (Control; 1 μg/ml). **(c)** Constitutively active (CA) or dominantly negative (DN) mutants of small GTPases were overexpressed in monolayer-cultured chondrocytes by adenoviral gene transfer, and adhesion to fibronectin-coated plates was evaluated. Results are mean ± standard error of the mean of four **(a)**, **(b)** or five **(c)** independent experiments, each in duplicate. **P <0.01.

To this end, we first conducted a cell attachment assay. Human articular chondrocytes were cultured in a monolayer for 2 or 7 days, and cell attachment was evaluated using noncoated plates or plates coated with BSA or fibronectin, a known ligand to α5β1 integrin. The result of this experiment showed that the attachment of chondrocytes to fibronectin-coated plates was obviously increased between 2 and 7 days after plating (Figure 3a). Next, to determine the significance of α5β1 integrin in cell attachment, 7-day cultured chondrocytes, once harvested, were incubated with a function blocking anti-α5β1 integrin antibody (JBS5) or control IgG for 90 minutes at room temperature, and were then plated onto fibronectin-coated plates. This experiment confirmed that the attachment of chondrocytes to fibronectin-coated plates was primarily mediated by α5β1 integrin (Figure 3b). Since the level of expression of α5ββ1 integrin changed little within that culture period (Additional file 4), this result was considered to indicate an increase in the activity of α5β1 integrin.

Given this result, we next examined whether RRAS is indeed involved in the observed increase in integrin activity. In the experiment, chondrocytes cultured in monolayers for 7 days were infected with the adenoviruses carrying CA or DN mutants of five small GTPases, and the attachment of the cells to fibronectin-coated plates was evaluated 3 days later. These five small GTPases are known to be involved in the regulation of integrin activity in certain types of cells [6,15-17]. In this experiment, cell attachment was significantly increased by the overexpression of a CA mutant of RRAS (CA-RRAS), and tended to be reduced by that of a DN mutant (DN-RRAS) (Figure 3c). Such a change in cell attachment was not observed with any other small GTPases.

### Induction of type I and type III procollagen expression and AKT phosphorylation was indeed regulated by RRAS in monolayer-cultured chondrocytes

**Figure 4:**
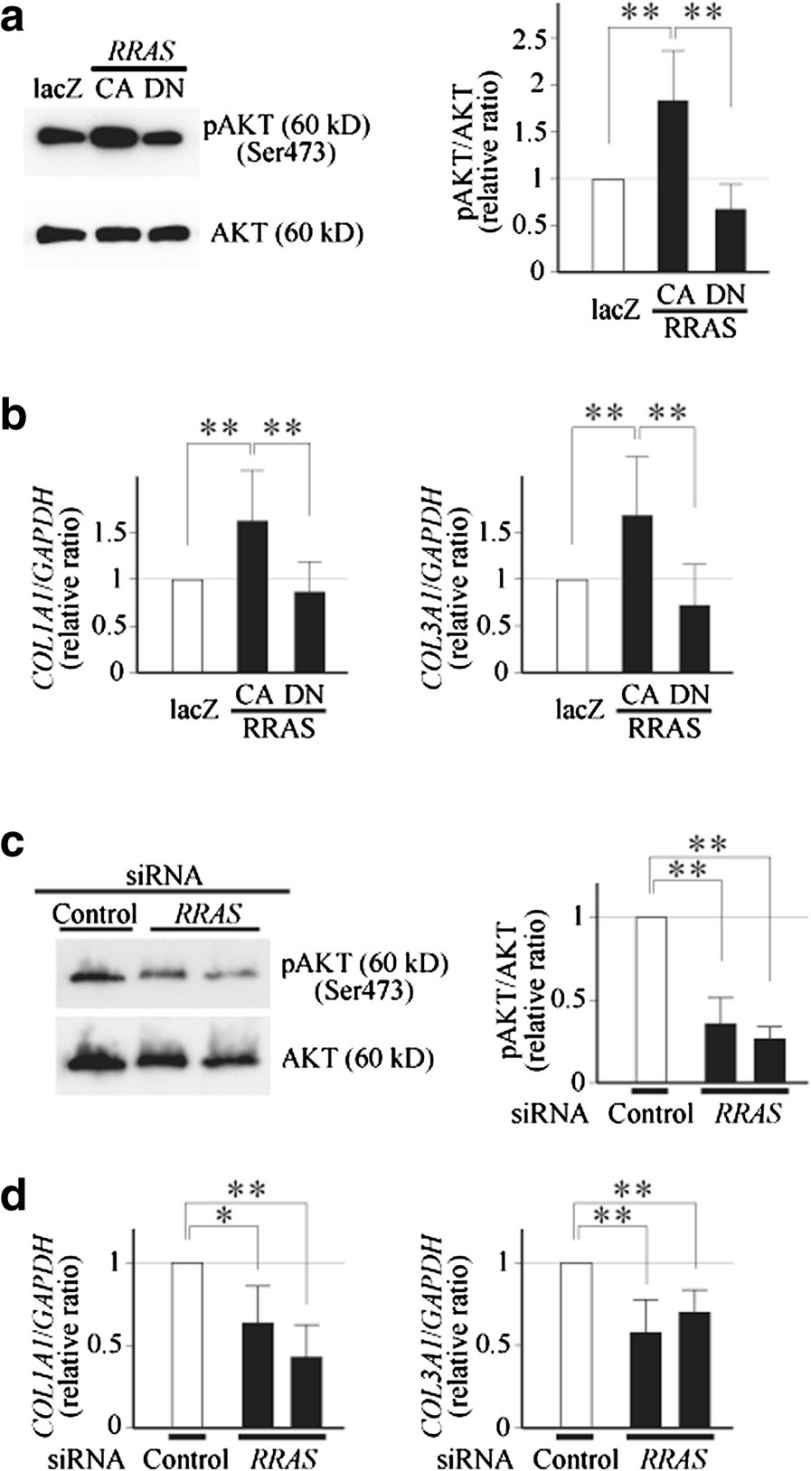
**RRAS may promote noncartilaginous procollagen gene expression by enhancing AKT phosphorylation. (a), (b)** lacZ or a constitutively active (CA) or dominantly negative (DN) mutant of RRAS was overexpressed in monolayer-cultured chondrocytes by means of adenoviral gene transfer. Three days later, phosphorylation of AKT at Ser473 was evaluated **(a)**, and expression of type I procollagen (*COL1A1*) and type III procollagen (*COL3A1*) was determined by quantitative PCR **(b)**. **(c), (d)** siRNA for RRAS (*RRAS*) or control siRNA was introduced into chondrocytes, and three days later, phosphorylation of AKT **(c)** and expression of indicated procollagen genes was evaluated (d). Two siRNAs for RRAS were used for the experiments. **(a)**, **(c)** Representative results of five independent experiments are shown. **(b)**, **(d)** Results are shown by relative ratios against values shown by open bars. Results are mean ± standard error of the mean of four **(d)** or six **(b)** independent experiments, each in triplicate. *P <0.05, **P <0.01. GADPH, glyceraldehyde 3-phosphate dehydrogenase.

The following experiments were performed to confirm the involvement of RRAS in the induction of type I and type III procollagen expression and AKT phosphorylation. If our above presumption is correct, phosphorylation of AKT should be modulated by RRAS through the change in the activity of α5β1 integrin. To examine this hypothesis, CA-RRAS or DN-RRAS was overexpressed in monolayer-cultured chondrocytes by means of adenoviral transduction, and phosphorylation of AKT was evaluated. As anticipated, the phosphorylation was enhanced by the overexpression of CA-RRAS, and tended to be reduced by that of DN-RRAS (Figure 4a). Consistently, in those chondrocytes, the expression of type I and type III procollagen was significantly elevated by the overexpression of CA-RRAS (Figure 4b). For further confirmation, we suppressed the expression of RRAS by RNAi and observed whether any changes occurred in AKT phosphorylation and noncartilaginous procollagen expression. In this experiment, AKT phosphorylation and procollagen expression were reduced, as predicted, by the suppression of RRAS expression (Figure 4c,d).

### Echistatin inhibited dedifferentiation of monolayer-cultured chondrocytes

**Figure 5:**
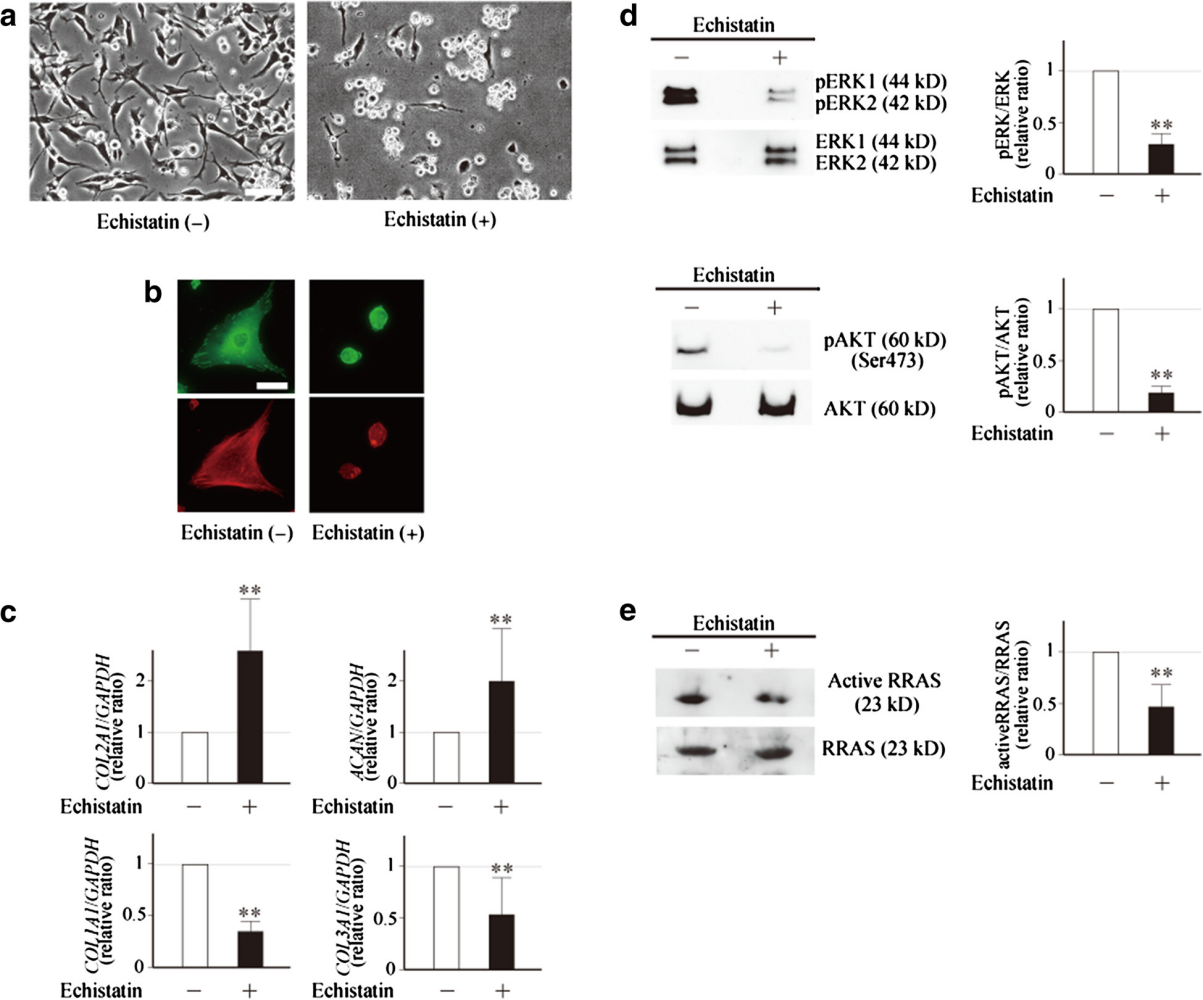
**Ehistatin inhibited dedifferentiation of monolayer-cultured chondrocytes. (a)** Primary cultured chondrocytes were cultured in monolayers in the presence or absence of echistatin, and cell morphology was observed 7 days after plating. Scale bar = 100 μm. **(b)** After macroscopic observation, immunofluorescent staining was performed to evaluate formation of focal adhesion and filamentous actin (F-actin) cytoskeleton. Green fluorescence indicates vinculin localized in focal adhesion, while red indicates phalloidine associated with F-actin. Scale bar = 20 μm. **(a)**, **(b)** Representative images of five independent experiments. **(c)** Chondrocytes were cultured in the presence or absence of echistatin for 7 days, and expression of type II procollagen (*COL2A1*) and aggrecan (*ACAN*) was evaluated by quantitative PCR, together with that of type I procollagen (*COL1A1*) and type III procollagen (*COL3A1*). Results are shown by relative ratios against the cells cultured without echistatin (open bars). Results are mean ± standard error of the mean (SEM) of five independent experiments, each duplicate. **P <0.01 against cells cultured without echistatin. **(d)** Chondrocytes were cultured for 7 days with or without echistatin, and phosphorylation of ERK and AKT was evaluated by western blot analysis using phosphospecific and then nonphosphospecific antibodies. Representative results of five independent experiments are shown together with those of densitometric measurement. For the latter, results are mean ± SEM. **(e)** Chondrocytes were cultured for 7 days with or without echistatin, and cell lysates were obtained. Amounts of active and total RRAS in the lysates were determined by western blot analysis with and without a pull-down assay, respectively. Experiments were repeated five times, and representative bands are shown together with those of densitometric measurement. For the latter, ratios of active RRAS against total RRAS are shown by mean ± SEM. Echistatin was used at 1 μM for these experiments. GADPH, glyceraldehyde 3-phosphate dehydrogenase.

From our current and previous observations, it is expected that dedifferentiation of chondrocytes could be prevented or minimized by the inhibition of engagement of α5β1 and αvβ5 integrins. We examined this possibility by experiments using echistatin, a disintegrin that potently inhibits ligation of ligands to various integrins [18,19]. The addition of echistatin to culture media obviously inhibited morphological change of the chondrocytes after plating (Figure 5a). Formation of focal adhesion and assembly of actin filament was strongly prevented by ehistatin (Figure 5b). Despite these changes, cell viability was not affected by the presence of echistatin in culture media (Additional file 5). Gene expression was then analyzed by quantitative PCR, and echistatin was known to prevent the decline of type II procollagen and aggrecan expression and the induction of type I and type III procollagen expression, which occurs in monolayer-cultured chondrocytes after plating (Figure 5c). Consistent with these results, phosphorylation of ERK and AKT was obviously reduced by the peptide (Figure 5d). Interestingly, the presence of echistatin in culture media also suppressed the activation of RRAS (Figure 5e), which has been shown to be elevated with the progression of dedifferentiation [6]. These results suggest the presence of a certain link between the engagement of integrins and activation of RRAS in articular chondrocytes.

### Echistatin improved quality of matrix synthesized by articular chondrocytes cultured in pellets

In cartilage tissue engineering, regeneration of cartilage matrix may be attempted with autologous chondrocytes [3-5]. In such a strategy, preservation of chondrocyte phenotype is a key to achieve successful tissue regeneration [20,21]. Since echistatin has been known to inhibit dedifferentiation of monolayer-cultured chondrocytes, we expected that this peptide could improve the quality of matrix synthesized by cultured chondrocytes. To examine this possibility, we cultured human articular chondrocytes in pellets for an extended period of 5 weeks, and investigated whether any changes occurred in gene expression or matrix synthesis by the presence of echistatin in the media. In this experiment, some pellets were cultured in the media containing CP4715, a synthetic compound that inhibits ligation of ligands to αvβ5 integrin [22,23], for comparison.

**Figure 6:**
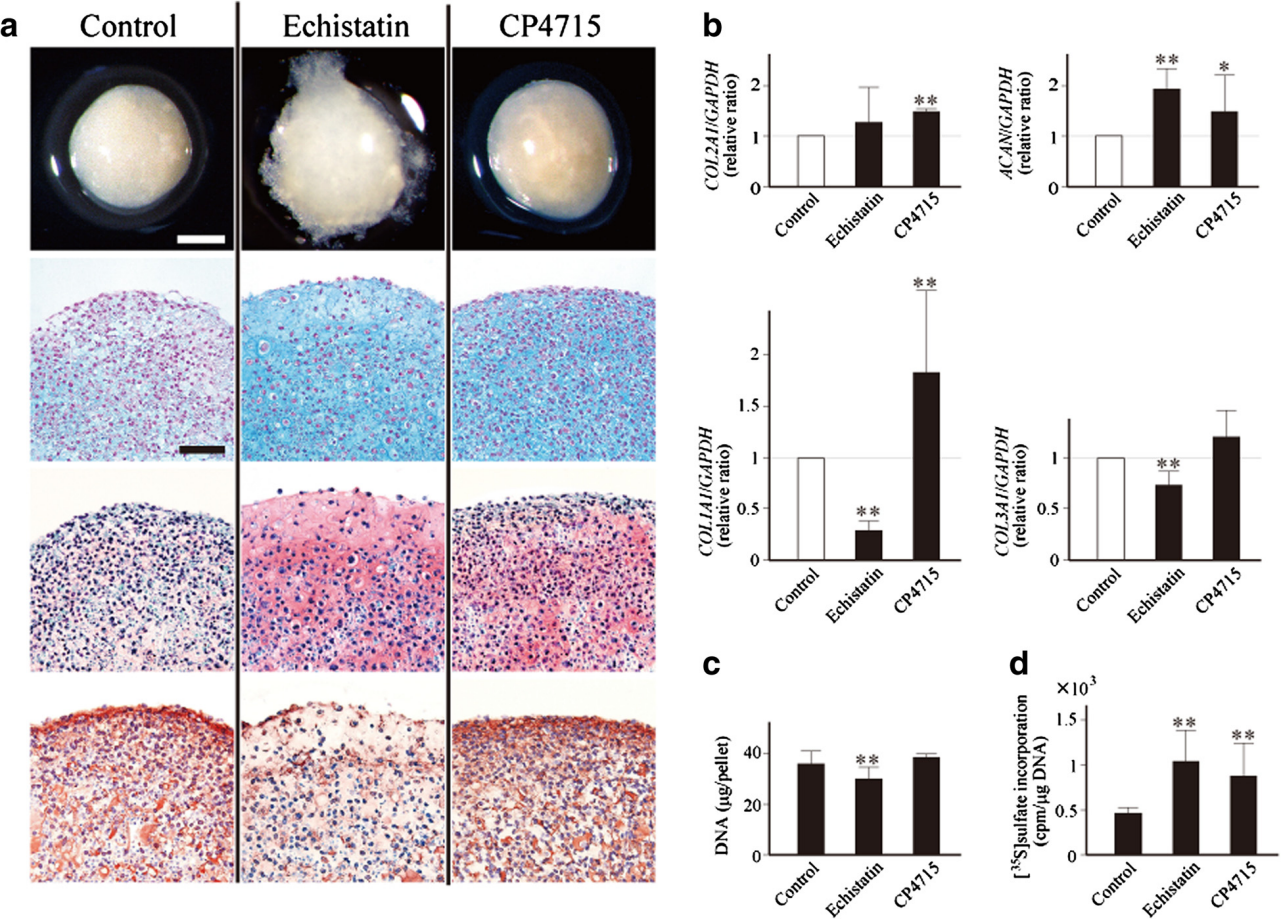
**Echistatin improved the quality of matrix synthesized by pellet-cultured chondrocytes.** Human articular chondrocytes were cultured in pellets in the media containing echistatin or CP4715, or in those containing none of them (Control). Pellets were cultured for 5 weeks in the presence or absence of echistatin or CP-4715. **(a)** Gross appearance (top row), photomicrographs of Alcian blue (second row) or Safranin O/fast green-stained sections of the pellets (third row) are shown together with those immunostained for type I collagen (bottom row). Scale bar = 1 mm (top row) or 200 μm (the other rows). **(b)** Expression of type II procollagen (*COL2A1*), aggrecan (*ACAN*), type I procollagen (*COL1A1*) and type III procollagen (*COL3A1*) in the pellets were evaluated by quantitative PCR. Results are shown by relative ratios against control pellets (Control; open bars). **(c)** DNA contents in the pellets. **(d)** Incorporation of [^35^S]sulfate into the pellets. Radioactivity was normalized by DNA content. For these experiments, echistatin and CP4715 were used at 1 μM and 100 nM, respectively. **(b)**, **(c)**, **(d)** Results are mean ± standard error of the mean of three to five pellets of four independent experiments. *P <0.05, **P <0.01 against control GADPH, glyceraldehyde 3-phosphate dehydrogenase.

In the pellets cultured without echistatin or CP4715 (control pellets), solid matrix with white and opaque appearance was synthesized by the chondrocytes (Figure 6a). In the pellets treated with echistatin, the matrix was much softer and more transparent. These echistatin-treated pellets had a frayed surface and tended to be larger in size, while the control pellets had a smooth surface and were smaller in diameter. The appearance of CP4715-treated pellets was close to that of the control pellets formed without echistatin, but the matrix tended to be softer and clearer, showing similarities to the echistatin-treated pellets.

In histology, the echistatin-treated pellets were known to contain an abundance of matrix. The matrix was intensely stained by Alcian blue and Safranin O, but was only weakly immunostained for type I collagen. Consistently, in those echistatin-treated pellets, the expression of aggrecan was enhanced, but the expression of type I and type III procollagen was reduced when compared with the control pellets (Figure 6b). Meanwhile, in CP4715-treated pellets, the expression of type II collagen and aggrecan was significantly increased, whereas the expression of type I and type III procollagen was not suppressed, or rather enhanced, probably due to the preference in integrin inhibition of this compound.

Although the echistatin-treated pellets contained fewer cells than the other pellets (Figure 6c), proteoglycan synthesis was the greatest with those pellets (Figure 6d), which was, again, consistent with the results of histological evaluation and gene expression analysis.

## Discussion

The results of this study indicated that α5β1 integrin could play a pivotal role in the induction of noncartilaginous procollagen expression in dedifferentiating chondrocytes. Previous studies have reported various roles of α5β1 integrin in chondrocytes. α5β1 integrin could be a mechanoreceptor for chondrocytes [24], and may regulate proliferation and survival of the cells [25,26]. α5β1 integrin may also promote catabolic responses in chondrocytes, inducing the expression of matrix metalloproteinases and proinflammatory cytokines [27,28]. Reactive oxygen species may be generated in chondrocytes upon the activation of α5β1 integrin [29]. In those catabolic responses, ERK, p38 mitogen-activated protein kinase, c-Jun N-terminal kinases, and protein kinase C pathways may be activated by this integrin [27,28].

Our current investigation has revealed another role of α5β1 integrin in articular chondrocytes to induce the expression of type I and type III procollagen. AKT signaling was considered to be involved in the induction. Although not known with chondrocytes, in fibroblasts, AKT signaling has been shown to induce the expression of type I procollagen [30-32]. With the progression of dedifferentiation, chondrocytes come to present a fibroblast-like phenotype. One might therefore reasonably consider that this reported role of AKT signaling in fibroblasts is acquired by cultured chondrocytes with the progression of dedifferentiation.

Current finding might explain a phenotypic change of chondrocytes observed *in vivo* with osteoarthritis. In this disease, chondrocytes undergo a phenotypic change similar to that observed during monolayer culture, and come to express type I and type III collagen abundantly [10,33,34]. This phenomenon has been known for decades, but the exact mechanism for this phenotypic change has not been determined. In osteoarthritis, chondrocytes come to produce fibronectin abundantly while it little exists in normal cartilage [35]. In osteoarthritic cartilage, fibronectin therefore probably accumulates around the chondrocytes, which would activate α5β1 integrin to induce the expression of type I and type III collagen. Although not demonstrated, we consider that this might be a pivotal mechanism for the phenotypic change of chondrocytes in osteoarthritis.

The results of this and our previous studies provide a comprehensive view of the dedifferentiation mechanism of chondrocytes. In monolayer-cultured chondrocytes, dedifferentiation may be promoted by α5β1 and αvβ5 integrins. These integrins seem to promote respective aspects of dedifferentiation. While α5β1 integrin may induce the expression of noncartilaginous procollagen gene expression via AKT signaling, αvβ5 integrin may suppress the expression of cartilage matrix genes through ERK signaling. The change in cell morphology may be promoted by αvβ5 integrin. Previously, those two integrins were shown to be dominant adhesion molecules that mediate the attachment of chondrocytes [36,37]. We now have shown that both of them not only are responsible for cell attachment but are also deeply involved in the metabolic and morphological changes that occur after plating.

In support of these proposed roles of integrins in dedifferentiation, inhibition of engagement of integrins by echistatin effectively prevented progression of dedifferentiation of monolayer-cultured and pellet-cultured chondrocytes (Figures 5 and 6). We have also confirmed that chondrogenic phenotype can be restored even in dedifferentiated chondrocytes that underwent subcultures, by the addition of echistatin to culture media (Additional file 6). As mentioned earlier, phenotypic change of the chondrocytes during culture is a critical issue in tissue engineering aiming to generate cartilage matrix by use of primary cultured chondrocytes [20,21]. Our current findings may provide a helpful hint for those attempting to restore impaired cartilage by this method.

Another important finding of our integrin studies is the pivotal role of RRAS in dedifferentiation. In the previous study, we determined that the activity of αvβ5 integrin is gradually increased by RRAS in the course of dedifferentiation [6]. In this work, we have revealed that RRAS also regulates the activity of α5β1 integrin. Based on these results, we now assume that the activation of RRAS could be a key event in chondrocyte dedifferentiation. RRAS is gradually activated in chondrocytes with the progression of dedifferentiation, and probably promotes phenotypic change of the chondrocytes by increasing the affinity and avidity of α5β1 and αvβ5 integrins to ligands.

Interestingly, this increase in RRAS activity during dedifferentiation may be inhibited by the inhibition of integrin engagement by echistatin (Figure 4d). Upon this finding, we currently assume the presence of a positive loop between integrin engagement and RRAS activation. Integrins could initiate the activation of RRAS when bound to ligands, which in turn might increase the avidity and affinity of these integrins to ligands, and thereby cause further integrin engagement. We think this mechanism might explain the prolonged time course of dedifferentiation in chondrocytes after plating.

In this study, we reported a pivotal role of α5β1 integrin in dedifferentiation of monolayer-cultured chondrocytes. Obviously, there are several limitations to this study. First, all experiments in this work were performed using chondrocytes prepared from osteoarthritic cartilage. The results might thus be affected by the phenotypic and metabolic change of the cells with the disease. Second, since most experiments were performed with primary cultured chondrocytes without subcultures, the influence of subculture has not been investigated. Third, although this and our previous studies have shown critical roles of integrins in dedifferentiation, the mechanism of dedifferentiation may not be fully elucidated, and some other mechanisms are possibly also involved in the process. Despite these limitations, our current findings are worth keeping in mind by anyone seeking a deeper understanding of the biology of articular chondrocytes.

## Conclusions

Articular chondrocytes undergo rapid dedifferentiation when cultured in monolayers. As dedifferentiation progresses, chondrocytes come to express type I and type III collagen abundantly. In this study, α5β1 integrin has been shown to promote the induction of this noncartilaginous procollagen expression through the activation of AKT signaling. In chondrocytes, the activity of α5β1 integrin may be regulated by RRAS, and thus RRAS could be a key molecule that regulates the process of dedifferentiation. We have also shown that the inhibition of integrin activation by echistatin, a potent disintegrin, effectively prevents dedifferentiation of monolayer-cultured chondrocytes, and improves the quality of matrix synthesized by pellet-cultured chondrocytes.

## Abbreviations

AKT: v-akt murine thymoma viral oncogene homolog; BSA: Bovine serum albumin; CA: Constitutively active; CDC42: Cell division cycle 42; DN: Dominantly negative; ERK: Elk-related tyrosine kinase; GST: glutathione S-transferase; HRAS: v-Ha-ras Harvey rat sarcoma viral oncogene homolog; mAb: Monoclonal antibody; MTT: 3-(4,5-dimethylthiazol-2-yl)-2,5-diphenyl tetrazolium bromide; PBS: Phosphate-buffered saline; PCR: Polymerase chain reaction; RAF1: v-raf-1 murine leukemia viral oncogene homolog 1; RNAi: Interfering RNA; RRAS: related RAS viral (r-ras) oncogene homolog; siRNA: Small interfering RNA.

## Competing interests

The authors declare that they have no competing interests.

## Authors’ contributions

HF and NF conceived the studies. NT and YI generated recombinant adenoviruses. HM performed histological evaluations. NT, TY and NF conducted all other experiments. TN, HF, ST, and NF analyzed the data. TN and NF wrote the manuscript with helpful comments from HF, HM, TN and ST. All authors read and approved the final version of the manuscript.

## Supplementary Material

Additional file 1: Table S1 Presenting sequence information for siRNAs used in the study. Click here for file

Additional file 2: Figure S1 Showing evaluation of cell viability after introduction of siRNA, an assay using MTT. siRNA for α5 (*ITGA5*) or β1 integrin gene (*ITGB1*) was introduced into primary cultured chondrocytes, and the cells were subjected to MTT assay 3 days after the introduction. A commercially available kit (Cell Proliferation Kit I; Roche Diagnostics) was used for the assay. Results are shown by relative ratios against cells given control siRNA. Three bars for each integrin gene indicate results of three siRNAs used for the gene. Results are mean ± standard error of the mean of three independent experiments, each in triplicate. GADPH, glyceraldehyde 3-phosphate dehydrogenase. Click here for file

Additional file 3: Figure S2 Showing that **(a), (b)** to specify the AKT isoform most involved in the induction of type I and type III procollagen expression during dedifferentiation, siRNAs for respective AKT isoforms were introduced into primary cultured chondrocytes, and 3 days later expression of type I (*COL1A1*) (a) and type III procollagen (*COL3A1*) (b) was evaluated by quantitative PCR. Results are shown by relative ratios against the cells given control siRNA (Control; (open bars). Two or three bars for each AKT gene indicate results of respective siRNAs used for the gene. Results are mean ± standard error of the mean of four independent experiments, each in duplicate. *P <0.05 and **P <0.01 against the cells given control siRNA. **(c)** RNA was extracted from 10 human osteoarthritic knee cartilages of 10 donors, and gene expression profiles were determined respectively by complementary DNA microarray analysis. Signal intensities for respective AKT isoforms are shown as mean ± standard error of the mean. For AKT3, tv1 and tv2 denotes transcript variant 1 (NM_005465) and 2 (NM_181690), respectively. GADPH, glyceraldehyde 3-phosphate dehydrogenase. Click here for file

Additional file 4: Figure S3 Showing that primary cultured articular chondrocytes were maintained in monolayers, and expression of α5 (*ITGA5*) **(a)** and β1 integrins (*ITGB1*) **(b)** was determined respectively 2 days (D2) and 7 days after plating (D7) by quantitative PCR. Expression levels at 7 days are shown by relative ratios against those at 2 days. **(c)** Expression of α5β1 integrin in monolayer-cultured chondrocytes was evaluated by flow cytometry at 2 days (shaded areas with fine lines) and 7 days (open areas with bold lines) after plating using a mAb for that integrin (10 μg/ml, HA5; Millipore). Representative results of five independent experiments are shown. **(d)** In flow cytometric analysis, fluorescence intensity for α5β1 integrin at 7 days (D7) was shown by relative ratio against that at 2 days (D2). **(a), (b), (d)** Results are mean ± standard error of the mean of five independent experiments. GADPH, glyceraldehyde 3-phosphate dehydrogenase. Click here for file

Additional file 5: Figure S4 Showing that primary cultured chondrocytes freshly prepared from cartilage tissues (P0) and the cells that underwent subculture twice (P2) were plated and cultured in the presence or absence of echistatin (1 μM). Cell viability was assessed 7 days after plating by MTT assay as described for Additional file 2. Results are shown by relative ratios against the cells cultured without echistatin. Results are mean ± standard error of the mean of three independent experiments, each in triplicate. Click here for file

Additional file 6: Figure S5 Showing that primary cultured chondrocytes were subcultured twice and then cultured in monolayers in the presence or absence of echistatin (1 μM). Seven days later, RNA was obtained, complementary DNA was synthesized, and expression of type II procollagen (*COL2A1*), aggrecan (*ACAN*), type I procollagen (*COL1A1*) and type III procollagen (*COL3A1*) were evaluated by quantitative PCR. Results are shown by relative ratios against the cells cultured without echistatin (open bars). Results are mean ± standard error of the mean of three independent experiments, each in triplicate. **P <0.01 against cells cultured without echistatin. GADPH, glyceraldehyde 3-phosphate dehydrogenase. Click here for file
