# Supplementary figures and images for: α5β1 integrin induces the expression of noncartilaginous procollagen gene expression in articular chondrocytes cultured in monolayers
Source: Arthritis Res Ther. 2013 Sep 19;15(5):R127. doi: 10.1186/ar4307 (PMC3978676; doi:10.1186/ar4307)

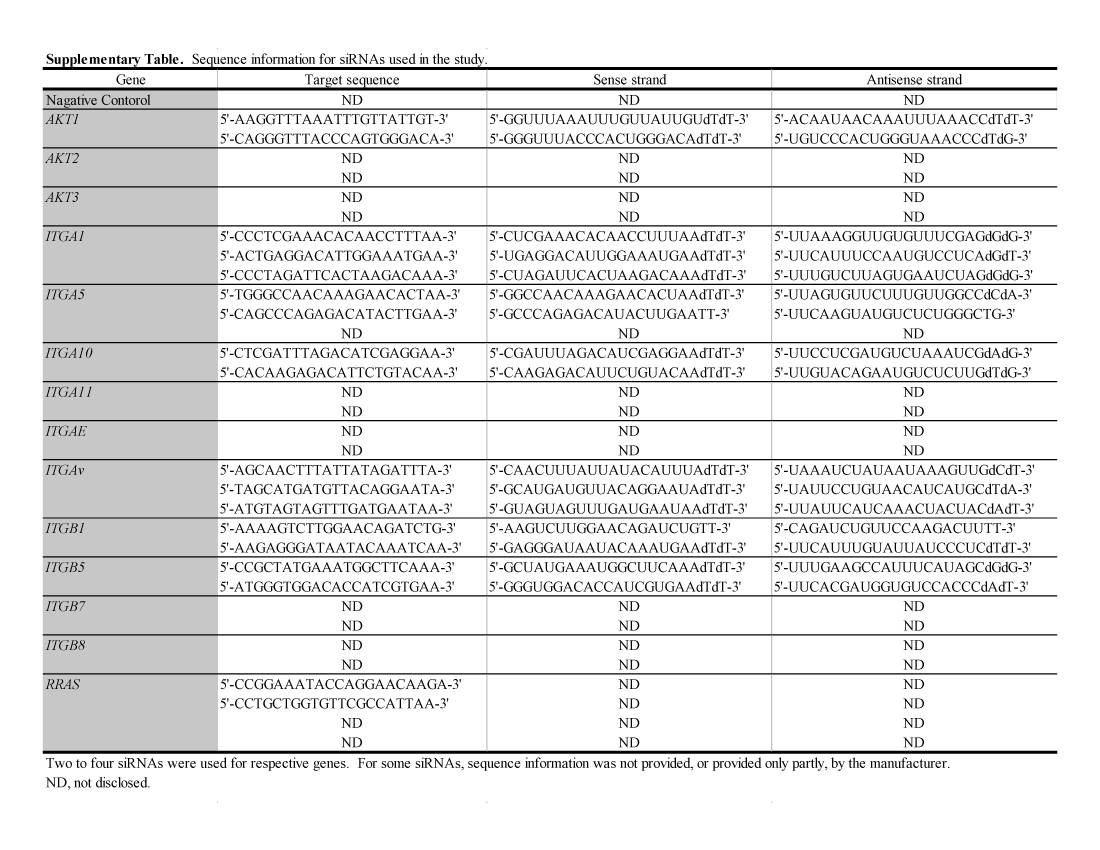

Supplement: Additional file 1: Table S1 — Presenting sequence information for siRNAs used in the study. [file ar4307-S1.tiff]

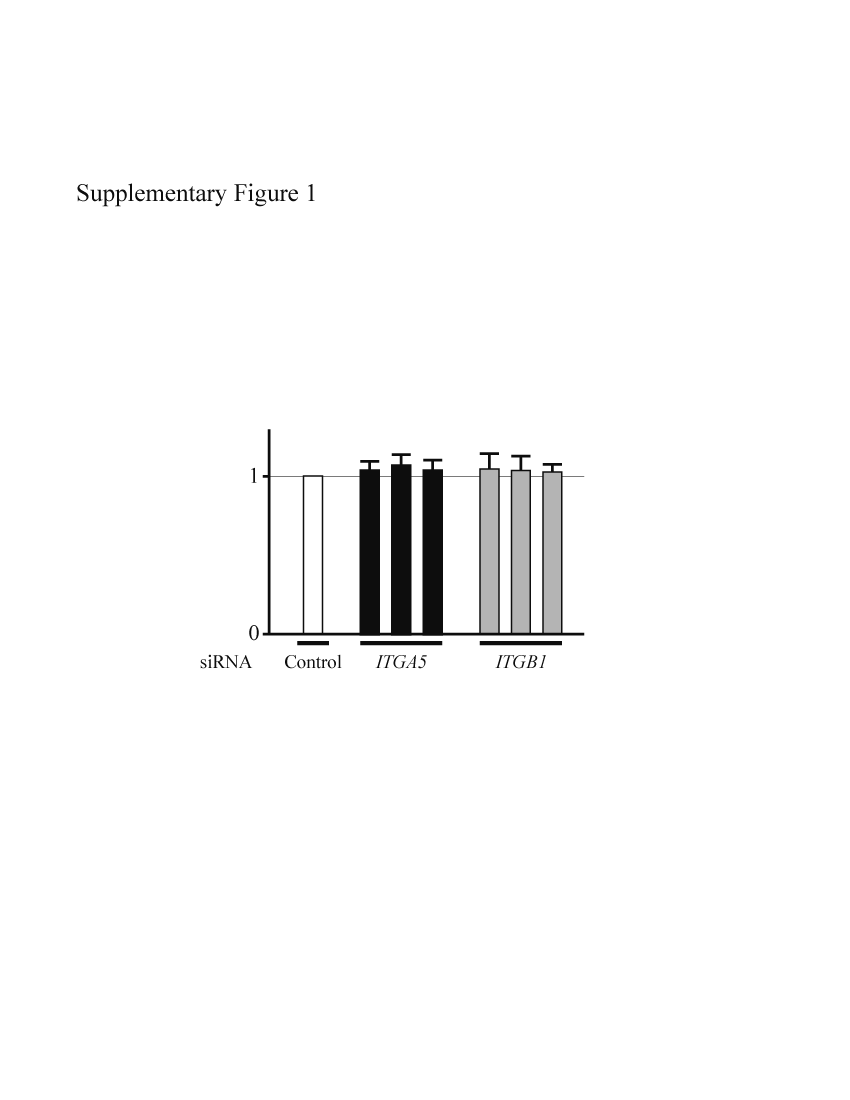

Supplement: Additional file 2: Figure S1 — Showing evaluation of cell viability after introduction of siRNA, an assay using MTT. siRNA for α5 (ITGA5) or β1 integrin gene (ITGB1) was introduced into primary cultured chondrocytes, and the cells were subjected to MTT assay 3 days after the introduction. A commercially available kit (Cell Proliferation Kit I; Roche Diagnostics) was used for the assay. Results are shown by relative ratios against cells given control siRNA. Three bars for each integrin gene indicate results of three siRNAs used for the gene. Results are mean ± standard error of the mean of three independent experiments, each in triplicate. GADPH, glyceraldehyde 3-phosphate dehydrogenase. [file ar4307-S2.tiff]

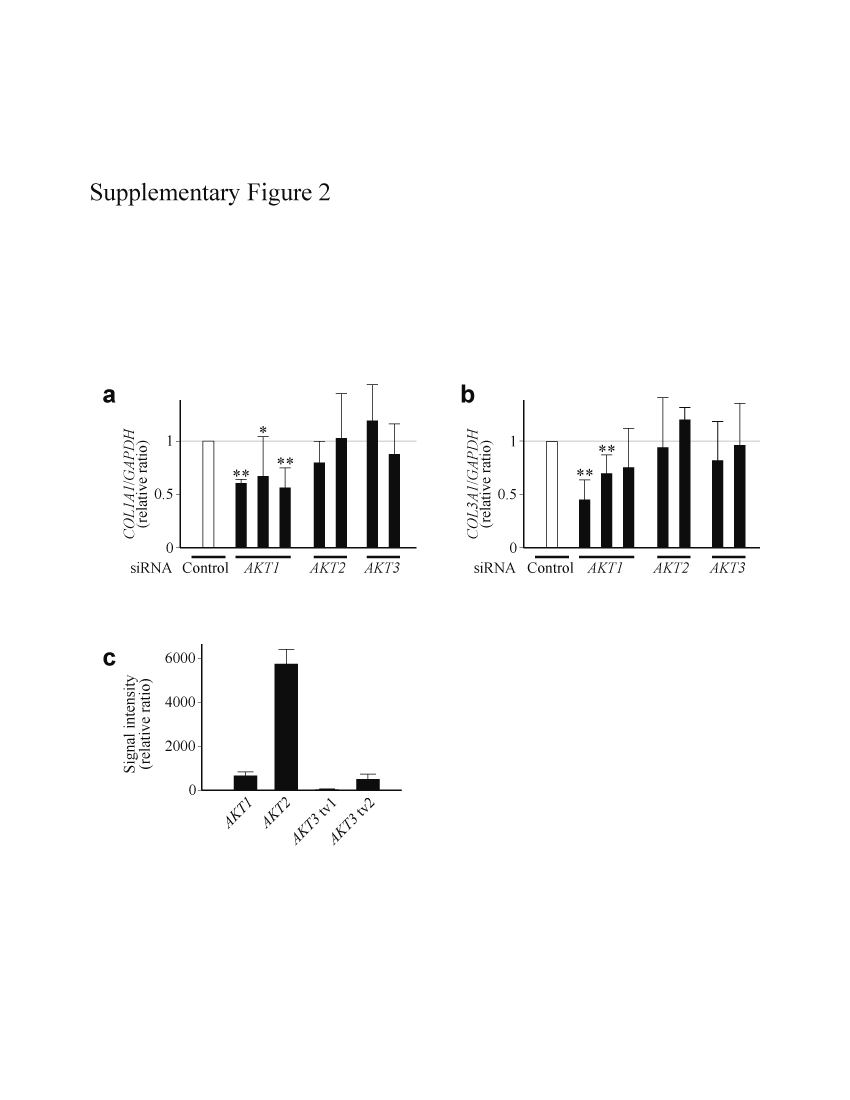

Supplement: Additional file 3: Figure S2 — Showing that (a), (b) to specify the AKT isoform most involved in the induction of type I and type III procollagen expression during dedifferentiation, siRNAs for respective AKT isoforms were introduced into primary cultured chondrocytes, and 3 days later expression of type I (COL1A1) (a) and type III procollagen (COL3A1) (b) was evaluated by quantitative PCR. Results are shown by relative ratios against the cells given control siRNA (Control; (open bars). Two or three bars for each AKT gene indicate results of respective siRNAs used for the gene. Results are mean ± standard error of the mean of four independent experiments, each in duplicate. *P <0.05 and **P <0.01 against the cells given control siRNA. (c) RNA was extracted from 10 human osteoarthritic knee cartilages of 10 donors, and gene expression profiles were determined respectively by complementary DNA microarray analysis. Signal intensities for respective AKT isoforms are shown as mean ± standard error of the mean. For AKT3, tv1 and tv2 denotes transcript variant 1 (NM_005465) and 2 (NM_181690), respectively. GADPH, glyceraldehyde 3-phosphate dehydrogenase. [file ar4307-S3.tiff]

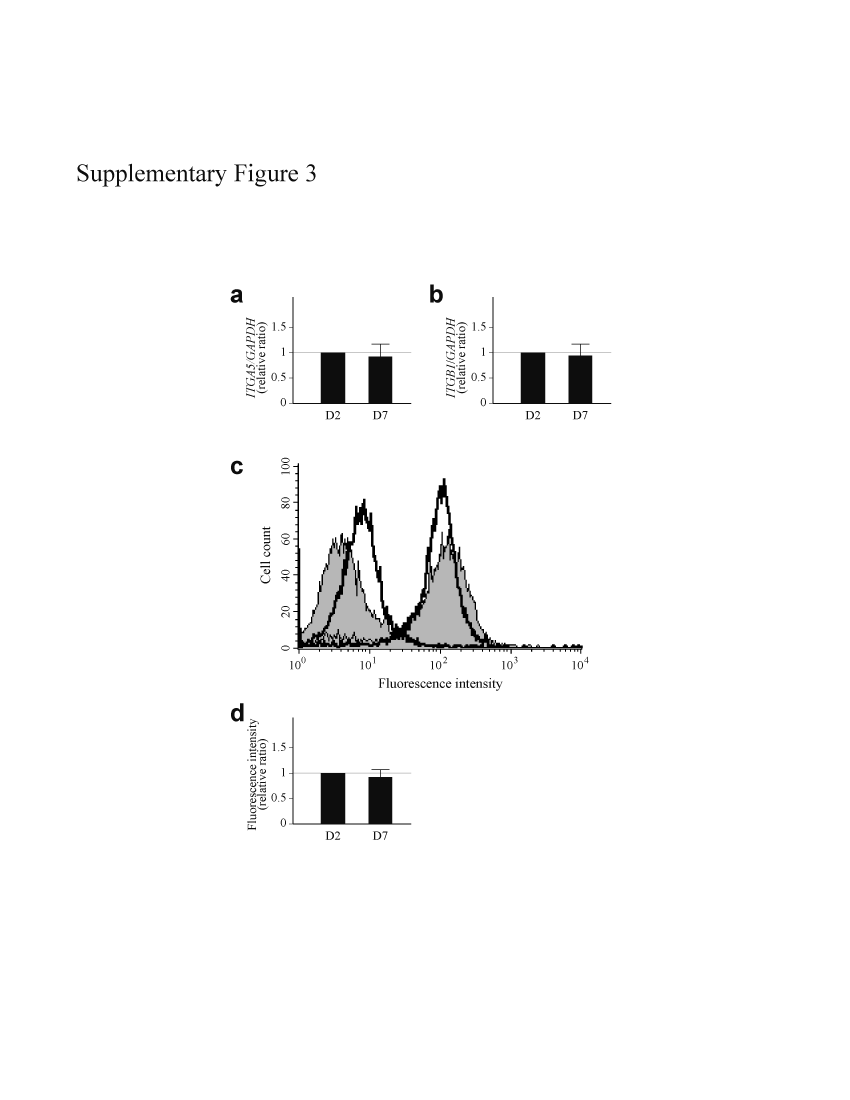

Supplement: Additional file 4: Figure S3 — Showing that primary cultured articular chondrocytes were maintained in monolayers, and expression of α5 (ITGA5) (a) and β1 integrins (ITGB1) (b) was determined respectively 2 days (D2) and 7 days after plating (D7) by quantitative PCR. Expression levels at 7 days are shown by relative ratios against those at 2 days. (c) Expression of α5β1 integrin in monolayer-cultured chondrocytes was evaluated by flow cytometry at 2 days (shaded areas with fine lines) and 7 days (open areas with bold lines) after plating using a mAb for that integrin (10 μg/ml, HA5; Millipore). Representative results of five independent experiments are shown. (d) In flow cytometric analysis, fluorescence intensity for α5β1 integrin at 7 days (D7) was shown by relative ratio against that at 2 days (D2). (a), (b), (d) Results are mean ± standard error of the mean of five independent experiments. GADPH, glyceraldehyde 3-phosphate dehydrogenase. [file ar4307-S4.tiff]

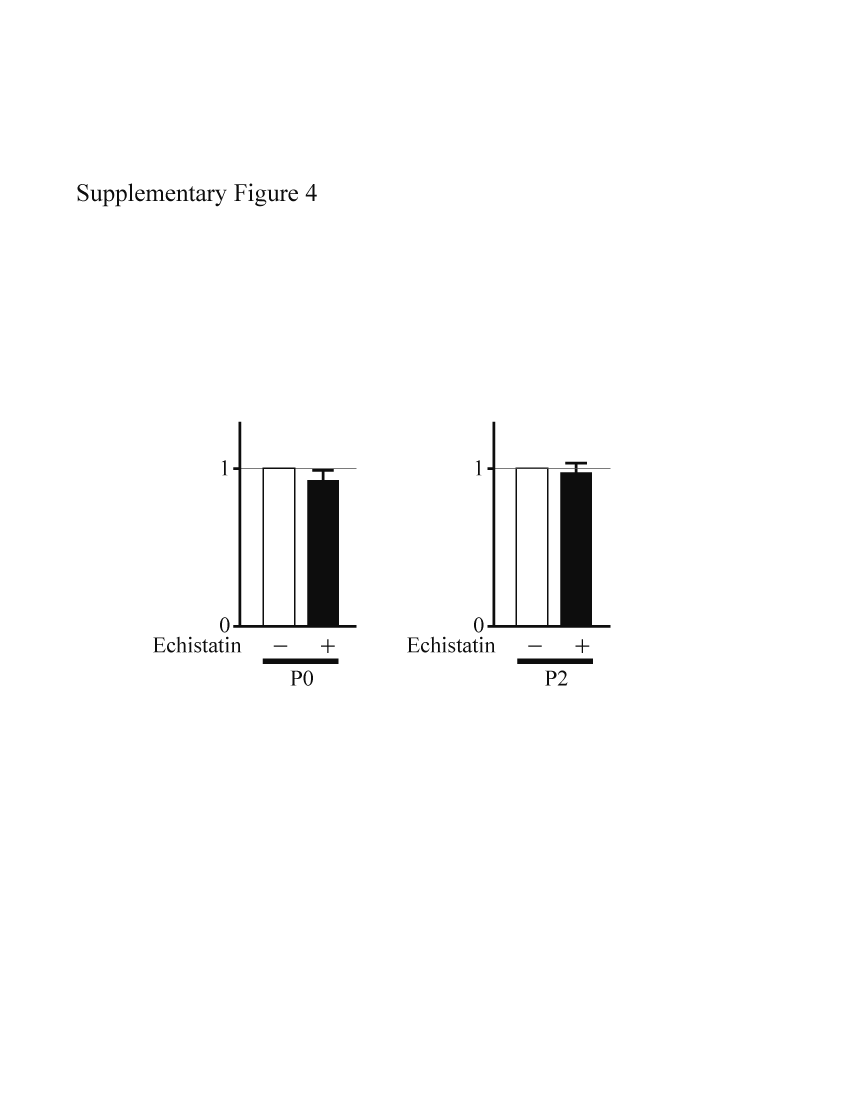

Supplement: Additional file 5: Figure S4 — Showing that primary cultured chondrocytes freshly prepared from cartilage tissues (P0) and the cells that underwent subculture twice (P2) were plated and cultured in the presence or absence of echistatin (1 μM). Cell viability was assessed 7 days after plating by MTT assay as described for Additional file 2. Results are shown by relative ratios against the cells cultured without echistatin. Results are mean ± standard error of the mean of three independent experiments, each in triplicate. [file ar4307-S5.tiff]

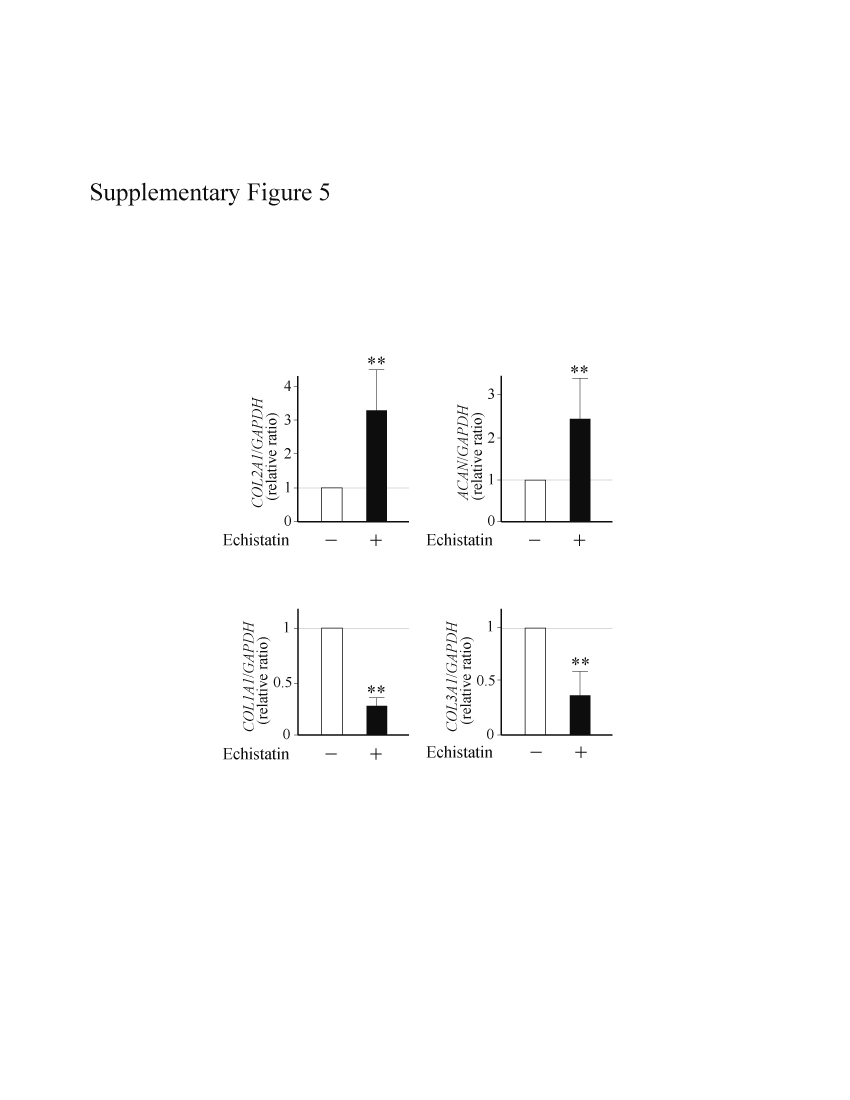

Supplement: Additional file 6: Figure S5 — Showing that primary cultured chondrocytes were subcultured twice and then cultured in monolayers in the presence or absence of echistatin (1 μM). Seven days later, RNA was obtained, complementary DNA was synthesized, and expression of type II procollagen (COL2A1), aggrecan (ACAN), type I procollagen (COL1A1) and type III procollagen (COL3A1) were evaluated by quantitative PCR. Results are shown by relative ratios against the cells cultured without echistatin (open bars). Results are mean ± standard error of the mean of three independent experiments, each in triplicate. **P <0.01 against cells cultured without echistatin. GADPH, glyceraldehyde 3-phosphate dehydrogenase. [file ar4307-S6.tiff]
